# Farewell

**DOI:** 10.1590/0100-3984.2022.55.6e1-en

**Published:** 2022

**Authors:** Edson Marchiori

**Affiliations:** 1 Editor-in-Chefe for Radiologia Brasileira.

For 21 years, I have had the honor of being Editor-in-Chief of the journal **Radiologia Brasileira (RB)**, the official publication of the Colégio Brasileiro de Radiologia e Diagnóstico por Imagem (CBR, Brazilian College of Radiology and Diagnostic Imaging), initially in partnership with Prof. Giovanni Guido Cerri and then on my own since 2011. It is now time for change and renewal. As of January, Prof. Valdair Muglia, the current president of the CBR, will become the new Editor-in-Chief of **RB**.

Over the years, the journal has undergone several changes, in attempts to improve it and adapt it to international standards. Initially, we instituted the online review system, which allowed greater agility in the processing of articles under evaluation, precluding the need to use traditional mail (which carries the risk of loss or misplacement), as well as allowing the articles to be evaluated by two reviewers and, occasionally, even by three. In addition, the journal now has an online version with full-text articles in English, allowing researchers from all over the world to access its content^(1)^.

The next goal was to get the journal indexed in international databases. That is the objective of practically all major national and international medical journals^(2)^. After **RB** came to be indexed in the Scopus/Scimago database, it began to be classified according to its impact^(3)^. PubMed Central subsequently approved the journal on the basis of scientific merit, paving the way for its final indexing in PubMed^(4)^.

Researchers in the field of radiology, especially those who participate in graduate (master’s or doctoral) programs evaluated by the Brazilian Office for the Advancement of Higher Education, have benefited greatly from these advances in the qualification of the journal, not only in the evaluation of the programs to which they belong but also in their personal evaluations, such as when they apply for research grants from funding agencies^(4)^.

The current goal, which has been sought with the indispensable participation of Prof. Muglia, is indexing in the Web of Science database (maintained by Clarivate Analytics), from which the Journal Citation Reports (JCR) is sourced annually. The JCR is the citation reference most widely used for calculating the impact factor of a journal^(5,6)^.

At this moment of farewell, some acknowledgments need to be made. Initially, I would like to thank the successive boards and presidents of the CBR for the trust placed in our work over all these years, during which they have supported and sponsored all of our initiatives. I would also like to thank Dr. Alexandre Moroz, the editorial coordinator of the journal, our right and left arm, who, with simplicity and competence, has been of fundamental importance in maintaining the quality of the journal. Without him, my task would have been impossible. The roles played by the editorial board and reviewers, who have been of enormous importance in the process, cannot be forgotten. Despite being extremely busy professionals, they have made part of their scarce free time available, working anonymously to review articles, evaluate them, edit them, accept them, or reject them, thus ensuring what is most important in a scientific journal: the quality and scientific rigor of the material published.

Although I am stepping away, I remain at the disposal of the CBR and the new Editor-in-Chief, Prof. Muglia, who has collaborated so much with the journal and with me personally in recent years, which undoubtedly makes him highly qualified to assume this important role.
